# Differences in anti-malarial activity of 4-aminoalcohol quinoline enantiomers and investigation of the presumed underlying mechanism of action

**DOI:** 10.1186/1475-2875-11-65

**Published:** 2012-03-08

**Authors:** Catherine Mullié, Alexia Jonet, Camille Desgrouas, Nicolas Taudon, Pascal Sonnet

**Affiliations:** 1 Laboratoire des Glucides, UMR-CNRS 6219, UFR de Pharmacie, 1 rue des Louvels, 80037 Amiens Cedex 1, France; 2 Laboratoire de Bioanalyse et Pharmacocinétique, UMR-MD3, Université de la Méditerranée, Institut de Recherche Biomédicale des Armées, Allée du Médecin Colonel Eugène Jamot, Parc du Pharo, BP 60109, 13262 Marseille Cedex 07, France

**Keywords:** *Plasmodium falciparum *, Anti-malarial activity, β-haematin, Quinoline, Enantiomer, Mefloquine

## Abstract

**Background:**

A better anti-malarial efficiency and lower neurotoxicity have been reported for mefloquine (MQ) (+)- enantiomer. However, the importance of stereoselectivity remains poorly understood as the anti-malarial activity of pure enantiomer MQ analogues has never been described. Building on these observations, a series of enantiopure 4-aminoalcohol quinoline derivatives has previously been synthesized to optimize the efficiency and reduce possible adverse effects. Their *in vitro *activity on *Plasmodium falciparum *W2 and 3D7 strains is reported here along with their inhibition of β-haematin formation and peroxidative degradation of haemin, two possible mechanisms of action of anti-malarial drugs.

**Results:**

The (*S*)-enantiomers of this series of 4-aminoalcohol quinoline derivatives were found to be at least as effective as both chloroquine (CQ) and MQ. The derivative with a 5-carbon side-chain length was the more efficient on both *P. falciparum *strains. (*R *)-enantiomers displayed an activity decreased by 2 to 15-fold as compared to their (*S*) counterparts. The inhibition of β-haematin formation was significantly stronger with all tested compounds than with MQ, irrespective of the stereochemistry. Similarly, the inhibition of haemin peroxidation was significantly higher for both (*S*) and (*R*)-enantiomers of derivatives with a side-chain length of five or six carbons than for MQ and CQ.

**Conclusions:**

The prominence of stereochemistry in the anti-malarial activity of 4-aminoalcohol quinoline derivatives is confirmed. The inhibition of β-haematin formation and haemin peroxidation can be put forward as presumed mechanisms of action but do not account for the stereoselectivity of action witnessed *in vitro*.

## Background

**Figure 1:**
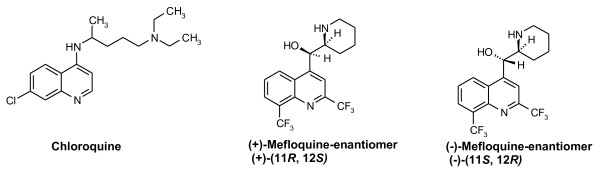
**Chloroquine and mefloquine enantiomers**.

Mefloquine (MQ) is a quinoline methanol derivative with a high schizontocide activity against *Plasmodium *species. This molecule possesses two asymmetric carbon atoms (Figure 1) and a long half-life (*circa *14 days) [1,2]. The latter propriety can be seen as a therapeutic advantage as a lower rate of relapses has been reported for anti-malarials with long half-lives [3]. Additionally, it also allows a weekly administration, making MQ a good candidate for long-term prophylactic treatments through improved compliance [4]. Despite an overall acceptable tolerability, dose-related neuropsychiatric adverse effects can occur [4,5], therefore, contraindicating MQ in individuals with a history of epilepsy or psychiatric disease. The opening of the piperidine ring at the 4-position of the quinoline scaffold was reported to yield molecules with a better potency and a lesser neurotoxicity than that of MQ [6]. The same team recently described a series of MQ non-piperidine analogs displaying a lower penetration in the brain than MQ along with a similar metabolic stability [7]. Another way to reduce neurotoxicity of MQ derivatives could be the synthesis of enantiomerically pure 4-aminoalcohol quinolines. Indeed, although MQ is commercially available as a racemic mixture of its *erythro *enantiomers, previous works showed that enantiomerism could play a part in the *in vitro *activity and toxicity of the drug. IC_50 _values for the (+)-enantiomer of MQ were found to be lower than those of the (-)-enantiomer by a factor of 1.6-1.8 on some strains (D6 and W2) [8] although no difference was found on other strains (L-3 and FCM29) [9]. As regards toxicity, the (-)-enantiomer was found to block to central nervous system adenosine receptors, while the (+)-enantiomer did not. This blockage of central adenosine receptors by the (-)-enantiomer is believed to result in neuropsychiatric symptoms associated with MQ [10].

With these differences in activity and toxicity in mind, a new enantioselective pathway to synthesize pure enantiomers of MQ amino-analogs was recently proposed [11,12]. In this paper, the *in vitro *anti-malarial activity on *Plasmodium falciparum *strains of a series of these enantiomers is reported as well as their effect on β-haematin formation and peroxidative-degradation of haemin, two mechanisms of action likely involved in the anti-malarial activity of 4-aminoquinolines [13].

## Methods

### Enantioselective synthesis of 4-aminoalcohol quinoline derivatives

All starting materials and reagents were obtained from commercial suppliers and were used without further purification. Reactions requiring anhydrous conditions were performed under a blanket of argon. All solvents were purified via literature procedures or used without further purification. 4-aminoalcohol quinoline derivatives synthesized are represented in Table 1. They were synthesized following the general procedure described by Jonet *et al. *[11,12].

**Table 1:** 4-aminoalcohol quinoline derivatives

R		
**Butyl**	(*R*)-**1**	(*S*)-**1**
**Pentyl**	(*R*)-**2**	(*S*)-**2**
**Hexyl**	(*R*)-**3**	(*S*)-**3**
**Heptyl**	(*R*)-**4**	(*S*)-**4**
**Octyl**	(*R*)-**5**	(*S*)-**5**
**Phenyl**	(*R*)-**6**	(*S*)-**6**

#### *Plasmodium falciparum *susceptibility assays

The *in vitro *activities our series of 4-aminoalcohol derivatives were tested over a concentration range of 0.78-400 nM against *P. falciparum *strains W2 and 3D7. These strains are resistant or susceptible to CQ, respectively. Additionally, W2 is sensitive while 3D7 displays a decreased susceptibility to MQ. The traditional labelled hypoxanthine method was used to assess anti-malarial activity, as described by Desjardins *et al. *[14]. CQ and MQ were routinely included as positive controls as well as negative controls using solvent. The resulting IC_50_s were calculated using Pk-Fit software [15]. Growth inhibition (I) and corresponding drug concentrations (C) were fitted according to a sigmoid model, described as:

$$\text{I}=\left({\text{I}}_{\text{max}}.{\text{C}}_{\gamma }\right)/\left({\text{C}}_{\gamma }+\text{ I}{{\text{C}}_{\text{5}0}}_{\gamma }\right)$$

where I_max _is the maximum growth inhibition and gamma the sigmoid factor of the curve.

### Inhibition of β-haematin aggregation

All chemicals were purchased from Sigma-Aldrich (Saint-Quentin Fallavier, France). The inhibition of β-haematin formation was tested using the technique by Baelmens *et al. *[16]. CQ and MQ were included in each series of experiments as controls. Briefly, 100 μL of a fresh 6.5 mM solution of haemin dissolved in 0.2 M NaOH was mixed with 100 μL of 3 M sodium acetate, 25 μL of 17.4 M acetic acid and 25 μL of the tested drug or relevant solvent as negative control. Stock solutions (20 mM) of CQ were prepared in water while MQ and 4-aminoalcohol quinoline derivatives were solubilised in MeOH/DMSO (4/1, v/v). Inhibition experiments were typically carried out over a concentration range of 0-2 mM (final concentration of the drug in wells). After a 24 h-incubation at 37°C under shaking, the supernatant resulting from centrifugation for 15 min at 3,300 g was discarded and the pellet washed with 200 μL DMSO. This latter step was repeated once and, after a final wash with water, the pellet was dissolved in 200 μL 0.1 M NaOH. After a further 1:30 dilution, absorption at 405 nm was read using a Multiskan EX multiplate reader (Thermo Labsystems, Issy les Moulineaux, France). Results are expressed as percentage of inhibition of β-haematin formation as compared to the relevant negative control result. Experiments were carried out at least in triplicate and, when achievable, IC_50_s were determined graphically.

### Inhibition of hydrogen peroxide-mediated haemin degradation

The monitoring of the peroxidative decomposition of haemin was carried out as previously described [17]. The reactions were performed either in sodium acetate 0.2 M (pH = 5.2) or HEPES 0.2 M (pH = 7.0). These values were chosen because the former is thought to be within the pH range of *P. falciparum *digestive vacuole and the latter within the pH range outside the food vacuole of the parasite. For inhibition assessments, stock solutions (1.2 mM) of CQ was prepared in the relevant buffer while stock solutions of MQ and 4-aminoalcohol quinoline derivatives were made in a mixture of MeOH/DMSO (4/1, v/v). The final concentration of drugs in wells was 100 μM. Additionally, to account for a possible shift in absorption due to the solvent, positive control curves were done using either water or MeOH/DMSO. The peroxidative reaction was initiated by the addition of 20 μL H_2_O_2 _(200 mM) and followed by measuring the decrease in absorption at the Soret band (405 nm) at 30 and 60 min using a Multiskan EX multiplate reader. Negative controls (with addition of H_2_O instead of H_2_O_2_) were also included in each experiment. Results are expressed as the percentage of unchanged haemin in the solution at the various time points.

### Statistical analysis

Each experimental condition was held in triplicate. Results are given as mean ± standard deviation of values obtained from at least three independent experiments.

Comparisons of percentages of β-haematin inhibition or remaining haemin (peroxidative degradation of haemin) were made using the Mann-Whitney signed rank test of Vassarstats website [18]. Statistical significance was defined as *p *< 0.05.

## Results

### *Plasmodium falciparum *susceptibility

IC_50_s for the various products are reported in Table 2. Interestingly, enantiomers with a (*S*)-absolute configuration were found to be more active than their (*R*)-counterparts by a factor ranging from two to 15-fold, according to the compound and the strain considered (Table 2). Compound (*S*)-**2 **was the more active of all, whatever the strain tested, followed by (*S*)-**3 **and (*S*)-**1**. Compounds (*S*)-**4**, (*S*)-**5 **and (*S*)-**6 **displayed similar IC_50_s, around 30 nmol/L. All (*S*)-enantiomers displayed lower IC_50_s than the racemic mixture of MQ, whatever the strain. Additionally, some IC_50 _values of (*S*)-enantiomers were also below or equivalent to those of CQ for the 3D7 strain.

**Table 2:** In vitro antimalarial activity of 4-animoalcohol quinoline enantiomers

Compound	IC_50_^a ^(nmol/L)		R/S ratio	
	
	W2	3D7	W2	3D7
Chloroquine	572 ± 112	25.7 ± 7.81	-	-

Mefloquine	26.5 ± 2.44	52.2 ± 4.18	-	-

(*R*)**-1**	ND^b^	27.8 ± 1.42	ND	2.19
		
(*S*)**-1**	ND	12.7 ± 0.67		

(*R*)**-2**	38.2 ± 3.63	74.7 ± 4.71	6.47	8.97
		
(*S*)**-2**	6.98 ± 0.62	8.33 ± 0.44		

(*R*)**-3**	142 ± 11.2	205 ± 16.2	15.1	14.1
		
(*S*)**-3**	9.40 ± 0.91	14.5 ± 1.23		

(*R*)**-4**	ND	254 ± 26.9	ND	7.70
		
(*S*)**-4**	ND	33.0 ± 1.55		

(*R*)**-5**	ND	290 ± 43.8	ND	9.32
		
(*S*)**-5**	ND	31.1 ± 0.93		

(*R*)**-6**	41.1 ± 9.62	104.5 ± 9.72	2.15	2.98
		
(*S*)**-6**	19.1 ± 4.03	35.1 ± 3.55		

^a^: Results expressed as mean ± standard deviation

^b^: Not Determined

#### Inhibition of β-haematin formation

Global and enantiomer specific percentages of inhibition are reported in Table 3. All 4-aminoalcohol quinoline derivatives displayed a significantly higher inhibition of β-haematin formation than MQ while CQ was significantly more efficient at inhibiting the process than all other compounds tested. Without reference to the absolute carbon configuration, compounds **4 **and **5 **were more potent than all the others (p < 0.002 and p < 0.02, respectively). No statistically significant differences were found between the different (*R*) or (*S*)-enantiomer 4-aminoalcohol quinoline derivatives except for compound **2 **for which the (*R*)-enantiomer was found to be slightly more active than its counterpart (p = 0.0477). β-haematin formation was significantly lower with compound (*R*)-**4 **than with all other compounds except for (*S*)-**4**, (*R*)-**5 **and (*S*)-**5**. Additionally, enantiomer (*S*)-**4 **had a significantly stronger inhibitory activity than (*R*)-**1**, (*S*)-**1**, (*S*)-**2**, (*R*)-**3 **and (*R*)-**6**.

**Table 3:** Global and enantiomer specific inhibition of β-haematin aggregation

Compound	Inhibition (%)	Enantiomer	Inhibition (%)	IC_50 _(mM)	CQ index^a^
Chloroquine	77.8 ± 6.27^b^	-	NA^c^	0.85 ± 0.11	-

Mefloquine	6.1 ± 4.63	-	ND^d^	> > > 2	> > > 2.35

**1**	47.1 ± 7.84	(*R*)	49.8 ± 7.54	> 2	> 2.35
		
		(*S*)	44.7 ± 7.58	> 2	> 2.35

**2**	49.1 ± 6.23	(*R*)	51.2 ± 7.03	1.90 ± 0.26	2.23
		
		(*S*)	46.5 ± 4.1	> 2	> 2.35

**3**	50.2 ± 5.05	(*R*)	48.3 ± 3.68	> 2	> 2.35
		
		(*S*)	51.8 ± 5.60	1.90 ± 0.21	2.23

**4**	59.3 ± 6.27*	(*R*)	60.8 ± 6.44	1.61 ± 0.17	1.89
		
		(*S*)	57.8 ± 6.07	1.29 ± 0.13	1.52

**5**	57.6 ± 8.31**	(*R*)	53.6 ± 8.89	1.86 ± 0.31	2.19
		
		(*S*)	61.7 ± 5.48	1.77 ± 0.16	2.08

**6**	50.6 ± 7.63	(*R*)	49.0 ± 7.95	> 2	> 2.35
		
		(*S*)	52.2 ± 7.38	1.92 ± 0.27	2.26

^a^: calculated as compound IC_50_/chloroquine IC_50_

^b^: Results expressed as mean ± standard deviation of the inhibition witnessed for each compound at a 2 mM concentration.

^c^: Not applicable

^d^: Not determined

*: Significantly higher than all other values in the same column except for chloroquine and compound **4 **(p ≤ 0.0011)

**: Significantly higher than values for mefloquine and compounds **1**, **2**, **3 **and **6 **(p < 0.02)

#### Inhibition of the peroxidative degradation of haemin

At pH 5.2, within the pH range of the food vacuole, unchanged haemin values were significantly lower for the MeOH/DMSO positive control after a 30 min incubation but not after 60 min, as compared to the water control (Table 4). When enantiomers sharing the same side-chain length were compared, no significant difference was witnessed. Compounds **2**, **3 **and **6 **were all more efficient in preventing haemin peroxidation than MQ, CQ and compounds **1**, **4 **and **5 **after a 30-min incubation, except for (*R*)-**2 **for which values were not statistically different from those of (*S*)-**5**. At 60 min, the same differences held (p < 0.0424) except for **2**, for which no difference was found with CQ activity, for (*S*)-**2**, for which values were similar to those of compounds **4 **and **5**, as were the values for compounds **5 **and **6**.

**Table 4:** Peroxidative degradation of haemin at pH 5

Compound		Unchanged haemin (%)	
		
		30 min	60 min
Negative control	Water	98 ± 6.0^a^	93 ± 5.9
	
	MeOH/DMSO	98 ± 1.0	97 ± 1.3

Positive control	Water	45 ± 7.0	36 ± 5.3
	
	MeOH/DMSO	41 ± 6.2*	33 ± 4.5

Chloroquine		53 ± 10.6	45 ± 8.5

Mefloquine		48 ± 9.1	39 ± 7.2

(*R*)**-1**		46 ± 7.3	39 ± 4.9

(*S*)**-1**		47 ± 7.8	40 ± 5.0

(*R*)**-2**		63 ± 2.8**	48 ± 3.6***

(*S*)**-2**		63 ± 4.1**	49 ± 3.8^†^

(*R*)**-3**		73 ± 2.4^‡^	60 ± 2.7^‡^

(*S*)**-3**		73 ± 2.0^‡^	59 ± 2.9^‡^

(*R*)**-4**		50 ± 12.7	45 ± 12.7

(*S*)**-4**		49 ± 12.1	45 ± 11.1

(*R*)**-5**		49 ± 11.1	44 ± 11.3

(*S*)**-5**		50 ± 13.5	45 ± 14.3

(*R*)**-6**		71 ± 2.6^$^	57 ± 3.9^£^

(*S*)**-6**		70 ± 4.6^$^	56 ± 4.5^£^

^a^: results given as mean ± standard deviation

*: significantly lower values than water positive control ones at the same time point (p ≤ 0.0124)

Significantly higher values at the same time point than those of:

**: chloroquine, mefloquine and compounds (*R*)-**1**, (*S*)-**1**, (*S*)-**4**, (*R*)-**4**, (*R*)-**5**, (*S*)-**5 **(p ≤ 0.0357)

***: mefloquine and compounds (*R*)-**1**, (*S*)-**1**, (*R*)-**4**, (*S*)-**4**, (*R*)-**5**, (*S*)-**5 **(p ≤ 0.0357)

^†^: mefloquine and compounds (*R*)-**1**, (*S*)-**1 **(p ≤ 0.0012)

^‡^: chloroquine, mefloquine and all other compounds but (*R*)-**6 **and (*S*)-**6 **(p ≤ 0.0006)

^$^: chloroquine, mefloquine and compounds (*R*)-**1**, (*S*)-**1**, (*R*)-**4**, (*S*)-**4**, (*R*)-**5**, (*S*)-**5 **(p **≤ **0.0025)

^£^: chloroquine, mefloquine and compounds (*R*)-**1**, (*S*)-**1**, (*R*)-**4**, (*S*)-**4 **(p ≤ 0.0424)

Within the more efficient compounds, no significant difference of activity was found between compounds **3 **and **6 **while compound **2 **showed a better protection of haemin against peroxidation than both compounds **3 **(p ≤ 0.0006) and **6 **(p ≤ 0.0151).

At pH 7.0, results were close to those witnessed at pH 5.2 [see Additional file 1]. The significant difference between MeOH/DMSO and water positive controls persisted throughout the 60-min incubation. Once more, no significant difference was found between enantiomers sharing the same side-chain and 4-aminoalcohol quinoline derivatives were generally more efficient than MQ and CQ at inhibiting this haemin degradation pathway. Similarly to pH 5.2, compounds **2**, **3 **and **6 **were the more efficient.

## Discussion

The *in vitro *anti-malarial activity of this series of 4-aminoalcohol quinoline enantiomers is reported here for the first time. Previously, only the activity of a racemic mixture of compound **1 **has been described [7]. It was reported to have an IC_90 _of 32 nmol/L on *P. falciparum *strain TM90C2A, resistant to CQ and MQ. In the present work, the activities of compounds (*R*)-**1 **and (*S*)-**1 **were evaluated on *P. falciparum *strain 3D7, displaying a reduced susceptibility to MQ (IC_50 _above 50 nmol/L). Compound (*S*)-**1 **was found to have an IC_50 _of 12.7 nmol/L while (*R*)-**1 **displayed a two-fold higher IC_50_. Corresponding IC_90_s for (*S*)-**1**, (*R*)-**1 **and MQ were respectively 36.0, 55.3 and 90.0 nmol/L, which is consistent with the previous report. However, the best anti-malarial activity in this series was witnessed for compound (*S*)-**2**, carrying a side-chain with 5 carbons, both on 3D7 and W2 strains, followed by compounds (*S*)-**1 **and (*S*)-**3**. Interestingly, (*S*)-enantiomers were found to be more active on both W2 and 3D7 *P. falciparum *strains than their counterparts, whatever the side-chain length. The (*S*)-enantiomer of MQ has previously been described as having a better *in vitro *activity than the (*R*)-one by a ratio close to 2-fold [8]. The results obtained with our series of 4-aminoalcohol quinolines indicate that this ratio would at least be of 2-fold and could reach up to 15-fold. These results also further back up the prominence of the absolute carbon configuration in the *in vitro *activity of this type of chemical structures. However, further tests on additional *P. falciparum *strains or isolates would help ascertain this prominence and its magnitude, as another study previously cast doubt on the impact of enantiomerism on the *in vitro *anti-malarial activity of MQ [9].

The molecular mechanism of action of MQ and 4-aminoalcohol quinolines is still poorly understood [13]. Some authors have suggested that the mechanism of action of these molecules could be similar to that of CQ [19,20]. To try to understand the mechanism underlying the enantiomeric specificity of action, we therefore chose to focus on investigating the possible inhibition of haem detoxification by 4-aminoalcohol quinolines. Indeed, compounds like CQ are thought to act through the inhibition of β-haematin formation, leading to a toxic accumulation of haem (ferriprotoporphyrin IX) in the parasite's food vacuole [21]. Alternatively, inhibition of the peroxidative degradation of haemin has also been proposed as a possible additional mechanism of action for quinolines and other molecules with anti-malarial properties [22,23]. Nevertheless, it has to be kept in mind that, despite a closely related structure, MQ has been described as less efficient in inhibiting β-haematin formation [23,24]. All compounds described in this paper displayed a better inhibitory activity on β-haematin formation than that of MQ, whatever the enantiomer and the side-chain length. However, these activities were significantly lower than that of CQ. Therefore, the better *in vitro *activity of our compounds as compared to CQ, cannot be accounted for by this sole mechanism of action. An interesting point to underline is that the longer the side-chain, the better the inhibitory activity was on this degradation pathway. Compounds **4 **and **5 **were indeed the more efficient in inhibiting β-haematin formation but were less active *in vitro *than compounds **1**, **2 **or **3 **on *P. falciparum *strains. Additionally, the results obtained on this pathway do not account for the discrepancies between the *in vitro *activity of 4-aminoalcohol quinoline enantiomers, as no significant difference was found in the activity of enantiomers with the same side-chain length. Stereochemistry is therefore unlikely to hinder the linkage of these compounds to the ferriprotoporphyrin IX structure, which is thought to be a pre-requisite for a good inhibition of β-haematin formation [25,26].

As regards the peroxidative degradation of haemin, no difference in the activity of enantiomers sharing the same side-chain length was once more witnessed. Moreover, no major difference between results obtained at pH 5.2 and 7.0 were highlighted. This is consistent with the fact that all of the tested molecules would mainly be monoprotonated at either pH, as all of them display calculated pKa_1 _values below 2.0 and pKa_2 _values above 7.8. Therefore, their reactivity would be the same inside as outside the food vacuole. Nevertheless, the most efficient compounds were compounds **2**, **3 **and **6 **this time. Hence, inhibition of haemin peroxidation results display a better correlation with the anti-malarial activity witnessed *in vitro *than the one observed with β-haematin.

On the whole, although this series of 4-animoalcohol quinoline derivatives yielded better results than MQ on the possible mechanisms of action investigated in this paper, these mechanisms did not provide a rationale for the enantiomer-dependent *in vitro *activity of our compounds. Another explanation, not necessarily exclusive of the mechanisms of action explored above, has to be sought to explain the selective *in vitro *activity of *S*-enantiomers. Such a hypothesis could be (i) another (additional) target of action in the parasite like the inhibition of an enzyme of *P. falciparum *, as stereoselectivity has previously been shown as important for some enzymatic interactions [27,28] or (ii) a differential active transport mechanism that would preferentially allow the passage of one of the enantiomers at the site of action of our molecules. The latter hypothesis sounds attractive as *P. falciparum *protein Pgh-1 (also termed PfMDR1), belonging to the family P-glycoprotein transporters and encoded by *pfmdr1 *, is mainly present on the food vacuole membrane and thought to transport anti-malarial drugs such as MQ in the food vacuole where they inhibit the formation of β-haematin [29,30].

Alternatively, Pgh1 could also be the primary target of MQ and 4-aminoquinoline activities through a direct inhibition of its transporter function, which has been demonstrated for quinine and halofantrine [31]. This would explain the reduced susceptibility to MQ of strains overexpressing *pfmdr *1 put forward by some authors. However, the link between overexpression of *pfmdr *1 and MQ resistance remains debatable, as shown with case reports from Asian isolates [32,33]. As regards stereoselectivity, it has indeed been shown for another active transporter belonging to the same P-glycoprotein family and situated on the blood-brain barrier that MQ enantiomers displayed differential behaviours towards its activity as the (-)- 11*S*-2'*R *MQ was found to preferentially accumulate in the brain [34,35]. Therefore, if such a stereoselective mechanism existed for Pgh-1 transport of this series of compounds, the preferential accumulation of (*S*)-enantiomers in the food vacuole where they would inhibit haem detoxification could explain their better activity as compared to (*R*)-enantiomers.

## Conclusions

In this paper, some molecules in a series of enantiomerically pure 4-aminoalcohol quinoline derivatives were shown to display a stronger anti-malarial activity (within the nanomolar range) than MQ and/or CQ. Moreover, (*S*)-enantiomers were shown to be more potent than their counterparts by a 2-15-fold ratio. As regards their mechanism of action, both (*S*) end (*R*)-molecules were shown to more strongly inhibit β-haematin formation than MQ as well as the peroxidative degradation of haemin for some of them. The better *in vitro *anti-malarial activity of (*S*)-enantiomers could be accounted for by a stereoselective specific transport in the food vacuole of the parasite through Pgh-1 transporter. Further experiments are warranted to confirm this hypothesis.

## Abbreviations

CQ: Chloroquine; MQ: Mefloquine.

## Competing interests

The authors declare that they have no competing interests.

## Authors' contributions

CM carried out the β-haematin and peroxidation studies, performed the statistical analysis and drafted the manuscript. AJ carried out the synthesis of 4-aminoalcohol quinoline derivatives. CD performed the *Plasmodium falciparum *susceptibility assays. NT participated to the *Plasmodium falciparum *susceptibility assays and helped to draft the manuscript. PS participated in the design and coordination of the study and helped to draft the manuscript. All authors read and approved the final manuscript.

## Supplementary Material

Additional file 1 **Peroxidative degradation of haemin at pH 7.0**. The data provided represent the mean values (± SD) of the peroxidative degradation of haemin performed at pH 7.0 along with the statistical analysis. Click here for file

## References

[B1] Karbwang J White NJ Clinical pharmacokinetics of mefloquine Clin Pharmacokinet 1990 19 264 279 10.2165/00003088-199019040-00002 2208897

[B2] Brocks DR Mehvar R Stereoselectivity in the pharmacodynamics and pharmacokinetics of the chiral antimalarial drugs Clin Pharmacokinet 2003 42 1359 1382 10.2165/00003088-200342150-00004 14674788

[B3] Douglas NM Nosten F Ashley EA Phaiphun L van Vugt M Singhasivanon P White NJ Price RN *Plasmodium vivax *recurrence following falciparum and mixed species malaria: risk factors and effect of antimalarial kinetics Clin Infect Dis 2011 52 612 620 10.1093/cid/ciq249 21292666 PMC3060895

[B4] Schlagenhauf P Adamcova M Regep L Schaerer MT Rhein HG The position of mefloquine as a 21st century malaria chemoprophylaxis Malar J 2010 9 357 10.1186/1475-2875-9-357 21143906 PMC3224336

[B5] Taylor WR White NJ Antimalarial drug toxicity: a review Drug Saf 2004 27 25 61 14720085 10.2165/00002018-200427010-00003

[B6] Dow GS Heady TN Bhattacharjee AK Caridha D Gerena L Gettayacamin M Lanteri CA Obaldia N Roncal N Shearer T Smith PL Tungtaeng A Wolf L Cabezas M Yourick D Smith KS Utility of alkylaminoquinolinyl methanols as new antimalarial drugs Antimicrob Agents Chemother 2006 50 4132 4143 10.1128/AAC.00631-06 16966402 PMC1694001

[B7] Milner E McCalmont W Bhonsle J Caridha D Cobar J Gardner S Gerena L Goodine D Lanteri C Melendez V Roncal N Sousa J Wipf P Dow GS Anti-malarial activity of a non-piperidine library of next-generation quinoline methanol Malar J 2010 9 51 10.1186/1475-2875-9-51 20149249 PMC2833169

[B8] Karle JM Olmeda R Gerena L Milhous WK *Plasmodium falciparum *: role of absolute stereochemistry in the antimalarial activity of synthetic amino alcohol antimalarial agents Exp Parasitol 1993 76 345 351 10.1006/expr.1993.1042 8513873

[B9] Basco LK Gillotin C Gimenez F Farinotti R Le Bras J In vitro activity of the enantiomers of mefloquine, halofantrine and enpiroline against *Plasmodium falciparum* Br J Clin Pharmacol 1992 33 517 520 1524966 10.1111/j.1365-2125.1992.tb04081.x PMC1381440

[B10] Shepherd J Use of (+) mefloquine for the treatment of malaria 1998 International patent WO98/39003

[B11] Jonet A Dassonville-Klimpt A Da Nascimento S Léger JM Guillon J Sonnet P First enantioselective synthesis of 4-aminoalcohol quinoline derivatives through a regioselective SN2 epoxide opening mechanism Tetrahedron-Asymmetry 2011 22 138 148 10.1016/j.tetasy.2011.01.003

[B12] Jonet A Dassonville-Klimpt A Mullié C Taudon N Sonnet P Enantioselective synthesis method 4-aminoalcoholquinoline derivatives and the use European Patent 2011 N11154229

[B13] Foley M Tilley L Quinoline antimalarials: mechanisms of action and resistance and prospects for new agents Pharmacol Ther 1998 79 55 87 10.1016/S0163-7258(98)00012-6 9719345

[B14] Desjardins RE Canfield CJ Haynes JD Chulay JD Quantitative assessment of antimalarial activity in vitro by a semiautomated microdilution technique Antimicrob Agents Chemother 1979 16 710 718 394674 10.1128/aac.16.6.710 PMC352941

[B15] Farenc C Fabreguette JR Bressolle F Pk-fit: a pharmacokinetic/pharmacodynamic and statistical data analysis software Comput Biomed Res 2000 33 315 330 10.1006/cbmr.2000.1548 11017724

[B16] Baelmans R Deharo E Munõz V Sauvain M Ginsburg H Experimental conditions for testing the inhibitory activity of chloroquine on the formation of β-hematin Exp Parasitol 2000 96 243 248 10.1006/expr.2000.4558 11162377

[B17] Sonnet P Mullié C In vitro antimalarial activity of ICL670: a further proof of the correlation between inhibition of β-hematin formation and of peroxidative degradation of hemin Exp Parasitol 2011 128 26 31 10.1016/j.exppara.2011.01.018 21295029

[B18] Lowry R VassarStats: Website for Statistical Computation http://faculty.vassar.edu/lowry/VassarStats.html

[B19] Slater AFG Chloroquine: mechanism of drug action and resistance in *Plasmodium falciparum* Pharmacol Ther 1993 57 203 235 10.1016/0163-7258(93)90056-J 8361993

[B20] Egan TJ Hempelmann E Mavuso WW Characterisation of synthetic β-haematin and effects of the antimalarial drugs quinidine, halofantrine, desbutylhalofantrine and mefloquine on its formation J Inorg Biochem 1999 73 101 107 10.1016/S0162-0134(98)10095-8 10212997

[B21] Ginsburg H Ward SA Bray PG An integrated model of chloroquine action Parasitol Today 1999 15 357 360 10.1016/S0169-4758(99)01502-1 10461161

[B22] Loria P Miller S Foley M Tilley L Inhibition of the peroxidative degradation of haem as the basis of action of chloroquine and other antimalarials Biochem J 1999 339 363 370 10.1042/0264-6021:3390363 10191268 PMC1220166

[B23] Mullié C Jonet A Dassonville-Klimpt A Gosmann G Sonnet P Inhibitory effect of ursolic acid derivatives on hydrogen peroxide--and glutathione-mediated degradation of hemin: a possible additional mechanism of action for antimalarial activity Exp Parasitol 2010 125 202 207 10.1016/j.exppara.2010.01.016 20109452

[B24] Chou AC Fitch CD Control of heme polymerase by chloroquine and other quinoline derivatives Biochem Biophys Res Comm 1993 195 422 427 10.1006/bbrc.1993.2060 8363618

[B25] Dorn A Vippagunta SR Matile H Jacquet C Vennerstrom JL Ridley RG An assessment of drug-haematin binding as a mechanism for inhibition of haematin polymerisation by quinoline antimalarials Biochem Pharmacol 1998 55 727 736 10.1016/S0006-2952(97)00510-8 9586944

[B26] Kutter D Chibale K Egan TJ Linear free energy relationships predict coordination and π-stacking interactions of small molecules with ferriprotoporphyrin IX J Inorg Biochem 2011 105 684 692 10.1016/j.jinorgbio.2011.02.008 21450272

[B27] Clapés P Fessner WD Sprenger GA Samland AK Recent progress in stereoselective synthesis with aldolases Curr Opin Chem Biol 2010 14 154 167 10.1016/j.cbpa.2009.11.029 20071212

[B28] Wandhammer M Carletti E Van der Schans M Gillon E Nicolet Y Masson P Goeldner M Noort D Nachon F Structural study of the complex stereoselectivity of human butyrylcholinesterase for the neurotoxic V-agents J Biol Chem 2011 286 16783 16789 10.1074/jbc.M110.209569 21454498 PMC3089521

[B29] Rohrbach P Sanchez CP Hayton K Friedrich O Patel J Sidhu AB Ferdig MT Fidock DA Lanzer M Genetic linkage of *pfmdr *with food vacuolar solute import in *Plasmodium falciparum* EMBO J 2006 25 3000 3011 10.1038/sj.emboj.7601203 16794577 PMC1500988

[B30] Ferreira PE Holmgren G Veiga MI Uhlén P Kaneko A Gil JP PfMDR1: mechanisms of transport modulation by functional polymorphisms PLoS One 2011 6 e23875 10.1371/journal.pone.0023875 21912647 PMC3164660

[B31] Sanchez CP Rotmann A Stein WD Lanzer M Polymorphisms within PfMDR1 alter the substrate specificity for anti-malarial drugs in Plasmodium falciparum Mol Microbiol 2008 70 786 798 18713316 10.1111/j.1365-2958.2008.06413.x

[B32] Phompradit P Wisedpanichkij R Muhamad P Chaijaroenkul W Na-Bangchang K Molecular analysis of pfatp6 and pfmdr1 polymorphisms and their association with in vitro sensitivity in *Plasmodium falciparum *isolates from the Thai-Myanmar border Acta Trop 2011 120 130 135 10.1016/j.actatropica.2011.07.003 21777558

[B33] Pradines B Bertaux L Pomares C Delaunay P Marty P Reduced in vitro susceptibility to artemisinin derivatives associated with multi-resistance in a traveller returning from South-East Asia Malar J 2011 10 268 10.1186/1475-2875-10-268 21923936 PMC3185277

[B34] Baudry S Pham YT Baune B Vidrequin S Crevoisier C Gimenez F Farinotti R Stereoselective passage of mefloquine through the blood-brain barrier in the rat J Pharm Pharmacol 1997 49 1086 1090 9401943 10.1111/j.2042-7158.1997.tb06047.x

[B35] de Lagerie Barraud S Comets E Gautrand C Fernandez C Auchere D Singlas E Mentre F Gimenez F Cerebral uptake of mefloquine enantiomers with and without the P-gp inhibitor elacridar (GF1210918) in mice Br J Pharmacol 2004 141 1214 1222 10.1038/sj.bjp.0705721 15023856 PMC1574889

