# Supplementary material for: Differences in anti-malarial activity of 4-aminoalcohol quinoline enantiomers and investigation of the presumed underlying mechanism of action
Source: Malar J. 2012 Mar 8;11:65. doi: 10.1186/1475-2875-11-65 (PMC3314553; doi:10.1186/1475-2875-11-65)
Supplement: Additional file 1 — Peroxidative degradation of haemin at pH 7.0. The data provided represent the mean values (± SD) of the peroxidative degradation of haemin performed at pH 7.0 along with the statistical analysis. [file 1475-2875-11-65-S1.DOC]

**Table. Peroxidative degradation of hemin at pH 7.0**

| Compound | | Unchanged hemin (%) | |
| --- | --- | --- | --- |
|  |  | 30 min | 60 min |
| Negative control | Water | 96 ± 4.8^a^ | 94 ± 4.4 |
|  | MeOH/DMSO | 98 ± 1.7 | 97 ± 1.9 |
| Positive control | Water | 35 ± 4.4 | 27 ± 3.0 |
|  | MeOH/DMSO | 32 ± 4.7^*^ | 23 ± 3.0^*^ |
| Chloroquine | | 50 ± 6.0 | 40 ± 5.5 |
| Mefloquine | | 33 ± 7.9 | 24 ± 5.7 |
| **(*R*)-1** | | 46 ± 9.2 | 34 ± 4.3 |
| **(*S*)-1** | | 40 ± 7.1 | 29 ± 2.8 |
| **(*R*)-2** | | 60 ± 4.5^**^ | 49 ± 2.8^**^ |
| **(*S*)-2** | | 58 ± 4.7^**^ | 48 ± 1.9^**^ |
| **(*R*)-3** | | 68 ± 10.0^***^ | 58 ± 7.1^***^ |
| **(*S*)-3** | | 70 ± 7.7^***^ | 60 ± 6.7^***^ |
| **(*R*)-4** | | 47 ± 9.0 | 36 ± 4.8 |
| **(*S*)-4** | | 49 ± 5.8 | 36 ± 3.9 |
| **(*R*)-5** | | 40 ± 5.9 | 29 ± 1.7 |
| **(*S*)-5** | | 40 ± 4.7 | 30 ± 2.0 |
| **(*R*)-6** | | 65 ± 5.9^†^ | 56 ± 4.8^‡^ |
| **(*S*)-6** | | 67 ± 5.8^†^ | 57 ± 3.6^‡^ |

a: results given as mean ± standard deviation

^*^: significantly lower values than water positive control ones at the same time point (p≤0.0183)

Significantly higher values at the same time point than those of :

^**^: chloroquine, mefloquine, (*R*)-1, (*S*)-1, (*R*)-4, (*S*)-4, (*R*)-5, (*S*)-5 (p≤ 0.0105)

^***^: chloroquine, mefloquine, (*R*)-1, (*S*)-1, (*R*)-2, (*S*)-2, (*R*)-4, (*S*)-4, (*R*)-5, (*S*)-5 (p≤0.0271)

^†^: chloroquine, mefloquine, (*R*)-1, (*S*)-1, (*S*)-2, (*R*)-4, (*S*)-4, (*R*)-5, (*S*)-5 (p≤0.424)

^‡^: chloroquine, mefloquine, (*R*)-1, (*S*)-1, (*R*)-2, (*S*)-2, (*R*)-4, (*S*)-4, (*R*)-5, (*S*)-5 (p≤0.0027)
